# Preoperative predictors for outcomes after total hip replacement in patients with osteoarthritis: a systematic review

**DOI:** 10.1186/s12891-016-1070-3

**Published:** 2016-05-17

**Authors:** Stefanie N. Hofstede, Maaike G. J. Gademan, Thea P. M. Vliet Vlieland, Rob G. H. H. Nelissen, Perla J. Marang-van de Mheen

**Affiliations:** Department of Orthopaedics, Leiden University Medical Center, Albinusdreef 2, Leiden, 2333 ZA The Netherlands; Department of Clinical Epidemiology, Leiden University Medical Center, Albinusdreef 2, Leiden, 2333 ZA The Netherlands; Department of Medical Decision Making, J10-S, Leiden University Medical Center, P.O. Box 9600, Leiden, 2300 RC The Netherlands

**Keywords:** Osteoarthritis, Hip arthroplasty, Review, Predictors, Outcome

## Abstract

**Background:**

This systematic review examines which patient related factors influence functional and clinical outcomes after total hip arthroplasty (THA) in patients with hip osteoarthritis (OA).

**Methods:**

We performed a systematic review according to the PRISMA guidelines. We searched databases and trial registries for prospective studies including OA patients who underwent primary THA. Studies with preoperative measurements on predictors, with at least 1 year follow-up were included. Risk of bias and confounding was assessed for two domains: follow-up rate and looking at independent effects.

**Results:**

Thirty-five studies were included (138,039 patients). Only nine studies (29 %) had low risk of bias for all domains thus suggesting an overall low quality of evidence. Studies were heterogeneous in the predictors tested and in the observed directions of the associations. Overall, preoperative function (13 studies (37 %), 2 with low risk of bias) and radiological OA (6 studies (17 %), 1 with low risk of bias) were predictors with the most consistent findings. Worse preoperative function and more severe radiological OA were associated with larger postoperative improvement. However, these patients never reached the level of postoperative functioning as patients with better preoperative function or less severe radiological OA. For age, gender, comorbidity, pain and quality of life the results of studies were conflicting. For BMI, some studies (*n* = 5, 2 with low risk of bias) found worse outcomes for patients with higher BMI. However, substantial improvement was still achieved regardless of their BMI.

**Conclusion:**

There is not enough evidence to draw succinct conclusions on preoperative predictors for postoperative outcome in THA, as results of studies are conflicting and the methodological quality is low. Results suggest to focus on preoperative function and radiological osteoarthritis to decide when THA will be most effective. The present mapping of current evidence on the relationship between patient related factors and outcomes provides better information compared to individual studies and may help to set patient expectations before surgery. In addition, these findings may contribute to discussions on how to achieve the best possible postoperative outcome for specific patient groups.

**Trial registration:**

This systematic review was registered in Prospero, registration number RD42014009977.

**Electronic supplementary material:**

The online version of this article (doi:10.1186/s12891-016-1070-3) contains supplementary material, which is available to authorized users.

## Background

Total Hip Arthroplasty (THA) is an effective treatment for most individuals who suffer from pain and loss of function due to end stage symptomatic hip osteoarthritis (OA). Parallel to the rising prevalence of hip OA, surgery rates are rising as well [1–4].

THA should not be given too early since the longevity of a prosthesis is limited [5] and outcomes after revision THA are generally worse compared to primary THA. Furthermore, about 10–15 % of the patients is not satisfied after primary THA [6, 7]. Therefore, defined criteria to assess when patients will benefit most from surgery are clearly warranted, as it may sometimes be better to first optimize the patient’s preoperative condition. Current practice suggests that disease severity and timing of surgery vary largely among centers and countries [8, 9]. The development of defined criteria to assess which patients will benefit most from surgery would preferably be based on the best available evidence. Previous reviews on which predictors determine outcome after THA were conducted some time ago or mainly focused on patient characteristics such as age, gender, socio economic status (SES)/education and BMI [10, 11]. Other patient related factors, such as preoperative function, pain and quality of life, were not included. Providing such an overview may contribute to discussions on how to achieve the best possible postoperative outcome for specific patient groups.

Therefore, aim of this study is to conduct a systematic review examining which preoperative patient related factors influence functional and clinical outcomes after THA in OA patients.

## Methods

We performed a systematic review according to the PRISMA guidelines. This systematic review was registered in Prospero, registration number RD42014009977.

### Search strategy

A search strategy was composed together with a trained librarian (see Additional file 1). On PubMed, MEDLINE (Ovid version), EMBASE (Ovid version), Web of Science, The Cochrane Library, CENTRAL, and CINAHL articles were searched published up to August 8, 2014. The search strategy consisted of the AND combination of five concepts: osteoarthritis, hip replacement, predictive determinants, postoperative, and functional and clinical outcomes. All relevant keyword variations were used, not only those in the controlled vocabularies of the various databases, but the free text word variations of these concepts as well.

The search strategy was optimized for all databases, taking into account differences of the controlled vocabularies as well as database-specific technical variations (e.g., the use of quotation marks). Animal-only studies were excluded. Additional strategies were composed for PubMed to find (1) studies not focusing on OA, (2) studies on patient satisfaction or activities of daily living, and (3) studies with the word after instead of postoperative.

### Inclusion of articles

We included prospective studies among primary hip OA patients who underwent primary THA, with preoperative and postoperative measurements on functional or clinical outcomes and a follow-up of at least one year. If studies included both THA and TKA patients, we only extracted the THA data. Thus the results after THA had to be described separately. We included predictors that could be determined using standard tests or questions used in clinical practice (i.e. patient characteristics, radiological images, questionnaires or physical exams). These variables could be the focus of the study, or included as confounder or covariate.

Articles were excluded with metal-on-metal prostheses, osteotomies before THA, only including bilateral surgeries, more than 5 % of the patients had other diagnoses than primary OA (i.e. secondary OA or rheumatoid arthritis) or different diagnoses could not be stratified, or more than 5 % of the population had received a revision and could not be stratified from primary THA. Furthermore, we excluded articles when results for hip and knee OA could not be stratified, data were collected retrospectively (i.e. preoperative status assessed after surgery) or if no full text was available online, via our library or after mailing the authors. In addition, studies were excluded when baseline scores were not reported, which is important to interpret the postoperative outcomes. Only for adverse outcomes such as loosening or complications, this was not applicable therefore these studies were included.

### Selection of studies

Articles were selected in two steps. First, two researchers (SH and MG) independently excluded articles based on the title and/or the abstract. Second, one researcher (SH) excluded articles based on the full text. A second researcher (MG) checked whether selected articles met the inclusion criteria.

### Assessment of risk of bias in included studies

Risk of bias was assessed by one author (SH) and checked by a second author (MG). It is unclear from the literature which elements causing risk of bias in observational studies should be assessed. Therefore, we tailored the risk of bias assessment to our research question, focusing on study design features that could potentially bias the association between exposure and outcome. Risk of bias was thus assessed for the following domains:

- Follow-up rate: less than 20 % loss to follow-up at 1 year was considered to represent low risk of bias [12, 13]. For longer follow-up, we considered 10 % loss to follow-up extra for each additional year as low risk of bias. Since reasons for loss to follow-up/non-responders were often not reported, we counted all loss to follow-up regardless of the reason.
- Looking at independent effects: e.g. the use of a multivariable model in etiological studies or a prediction model. For example when adjustments in analyses were made for confounding factors (at least one), it was considered as low risk of bias.

When no consensus between the two review authors was reached, a third review author (PM) was consulted for the final decision.

### Data extraction

Data were extracted using a pre-defined data extraction form. Articles meeting the criteria were closely examined and data were extracted by one author (SH) and checked by a second author (MG). When no consensus could be reached, a third review author (PM) was consulted. We extracted the following information: sample size, gender, age, follow-up time, follow-up rate and adjustments in statistical analyses. Furthermore, we reported each predictor for all outcomes per study and their direction.

The following predictors were included:

- Patient characteristics: age, gender, SES/education, BMI
- Disease characteristics: radiological OA severity, comorbidities
- Patient expectations
- Pain
- Function
- Health related quality of life
- Mental well-being

All reported outcomes at different follow-up moments (≥1 year) for the above described predicting factors were extracted as reported in the included study. We examined both the change in outcome scores (postoperative score - preoperative score) and the level of the postoperative outcome, as patients with lower baseline scores are more likely to improve, but may not reach the same postoperative levels as patients with higher baseline scores.

Given the heterogeneity of predictors and outcomes, pooling of data using meta-analysis was not possible so that only descriptive analyses were conducted.

## Results

### Search

**Fig. 1:**
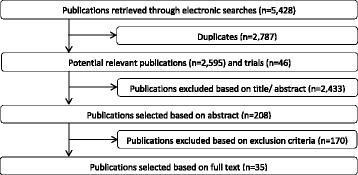
Flow diagram of included and excluded publications

The bibliographic databases yielded a total of 2,595 references and 46 additional studies in trial registers (Fig. 1). Full-text papers of 208 references were assessed for eligibility. We excluded 170 articles, mainly because more than 5 % of the population had a diagnosis other than primary OA or a revision surgery. Thirty-five studies fulfilled our inclusion criteria.

### Risk of bias

Table 1 shows that 14 studies (40 %) had low risk of bias for the follow-up domain. Eight studies [14–21] had a high risk of bias on this domain. One study [22] had a loss to follow-up of >20 % in the first and third year, but a low loss to follow-up at 5 and 7 years, so that risk of bias was unclear. Twelve studies had unclear risk of bias as the loss to follow-up was not described. Four of these studies were registry studies [23–26] and one study [27] was based on Medicare claims.

**Table 1:** Risk of bias and confounding

First author, year	Follow-up (years)	Follow-up (% missing)	Looking at independent effects
Bethge, 2010 [14]	1	28.9	Age, gender and self-efficacy expectations
Clement, 2011 [43]	1	ND	Age
Clement, 2011 [47]	1	ND	Age, SF-12 scores and length of stay
Cushnaghan, 2007 [34]	Mean 8.8	48 cases	Prediction model
		53 controls	
Davis, 2011 [35]	5	HHS: 28	Age, gender, operating consultant, and a diagnosis of cancer, atherosclerotic disease, cardiac disease, diabetes mellitus, osteoporosis and phlebitis
		SF-36: 32	
Duivenvoorden, 2013 [15]	1	31	Age, gender, time spent on waiting list and unbalanced characteristics between study population and patients lost to follow-up
Gandhi, 2010 [36]	Mean 3.3	14 at 1 year follow-up	Age, gender, BMI, SF-36 Mental Health (MH) scores, method of fixation (cemented vs uncemented), and comorbidity
Gordon, 2014 [37]	1	8	Age, gender, Charnley classification, previous contralateral THR, and preoperative pain VAS
Gordon, 2014 [38]	1	8	Gender, previous contralateral hip surgery, pain, and Charnley classification
Greene, 2014 [16]	1	66.7	Bayesian model averaging with age, gender, Charnley classification, presence of comorbidities, whether the included hip was the first or second in the time interval, marital status, and education level
Fortin, 2002 [39]	2	25.7^a^	Age, gender, education, and comorbidity
Haverkamp, 2013 [28]	Mean 2.3	18.6^a^	No
Heiberg, 2013 [17]	1	27.3	Prediction model
Ieiri, 2013 [49]	1 and 3	ND	Canonical correlation analysis
Johansson, 2010 [29]	2	ND	No
Judge, 2014 [19]	1	30.8	Age, sex, SF-36 mental health, comorbidities, fixed flexion, analgesic use, college education, OA in other joints, expectation of less pain, radiological K&L grade, ASA grade, years of hip pain
Judge, 2013 [40]	each year up to 5	20 at 1 year, 30 at 5 year	Multivariable model
Judge, 2012 [48]	Mean 8	61.3^b^	Prediction model
Judge, 2011 [18]	1	31.6	Age, sex, school education, ASA grade, K&L grade, BMI, medication use
Katz, 2012 [27]	12	ND	Patient age, sex, race, Medicaid eligibility, comorbidity and hospital and surgeon annual THA volume
Kennedy, 2011 [44]	Up to 1.3	ND	Age, gender, body mass index
Keurentjes, 2013 [20]	1.5–6	54.1^a^	Age, sex, Charnley Comorbidity Classification and BMI
McHugh, 2013 [41]	1	11.7	Multivariable model
Meding, 2000 [22]	Mean 2.7	11.4 at 1 year, 37.2 at 3 years, 64.8 at 5 years and 84.3 at 7 years	Age and gender
Nikolajsen, 2006 [30]	1–1.5	6.4	No
Nilsdotter, 2003 [42]	Mean 3.6	9.6	Multivariable model
Nilsdotter, 2002 [32]	1	16.2	No
Nilsdotter, 2001 [31]	1	11.9	No
Röder, 2007 [24]	Mean 4.3	ND (registry study)	Gender, age, and follow-up year
Rolfson, 2009 [23]	1	ND (registry study)	Age, gender and comorbidity
Sadr Azodi, 2008 [25]	3	ND (registry study)	Age at the time of surgery, calendar period, and fixation principle
Sarasqueta, 2012 [21]	1	29	Prediction model
Stickles, 2001 [26]	1	ND (registry study)	No
Street, 2005 [33]	1 and 2	ND	No
Tanaka, 2010 [45]	1	ND	Age, sex, changes in the LLD, vertical ATD, femoral offset, and the horizontal and vertical position of the center of the femoral head, stage of hip OA (advanced or terminal); HHS, and the duration of OA

*SF-12* 12-item Short Form Health Survey, *HHS* Harris Hip Score, *SF-36* 36-item Short Form Health Survey, *BMI* body mass index, *MH* mental health, *THR* total hip replacement, *VAS* visual analogue scale, *OA* osteoarthritis, *K&L grade* Kellgren-Lawrence, *ASA* American Society of Anesthesiologists, *LLD* leg length discrepancy, *ATD* articulotrochanteric distance

*ND* not described or partly described

^a^For patients with THA and TKA, not separately described

^b^At 6 months

Most studies (*n* = 28) adjusted for confounders or used a prediction model (low risk of bias), but differed from stratifying for one variable to multivariable adjustment (Table 1). Seven studies [26, 28–33] did not adjust for other factors in the analyses (high risk of bias).

Only nine studies (29 %) had low risk of bias across both domains: Cushnaghan [34], Davis [35], Gandhi [36], Gordon [37, 38], Fortin [39], Judge [40], McHugh [41], and Nilsdotter [42], to which we will refer as low risk of bias studies. Thus most studies had unclear or high risk of bias for least one domain, to which we will refer as high risk of bias studies, suggesting overall low quality of evidence.

### Study characteristics

The 35 included studies were all observational cohort studies. Table 2 shows that studies differ considerably in which factors predict outcomes after THA, given that only few significant associations were found per combination of a prognostic factor and outcome. Many studies assessed the effect of a prognostic factor on more than one outcome, as such it was possible to find a significant association for one outcome while no association with another outcome was found. As a result, a study may be described below both as a significant and a non-significant association. Most studies assessed outcomes through self-reported questionnaires and if the Harris Hip Score was used [29, 33] it was often not described who performed the physical examination. Additional file 2 shows the number of patients in each included study, the gender and age distribution, follow-up time, and significant associations observed. A total of 138,039 patients were included with average age from 60 to 84 years. Four studies used registry data [23–26] and one study used Medicare claims data [27]. The follow-up time varied from 1 year to a mean of 12 years [27].

**Table 2:** Number of reported outcomes for each prognostic factor

Prognostic factors	SF-36	EQ-5D	SF-12	WOMAC	OHS	HHS	Pain	Satisfaction	Walking distance	Revision	Dislocation	Other outcomes^a^
Age	5	1	1	3	2	-	-	1	1	1	-	Complications
Gender	3	3	-	-	-	-	1	2	1	1	-	
SES/education	-	1	-	1	1	-	1	1	-	-	-	
Comorbidities	4	3	-	1	1	-	1	1	-	-	2	
BMI	2	-	-	-	2	1	-	-	-	-	-	Superficial infection Ascending and descending stairs
Radiological OA severity	3	-	-	1	-	-	1	1	-	-	-	Gait improvement
Patient expectations	-	-	-	1	-	1	-	-	-	-	-	
Pain	3	-	-	4	-	1	1	-	-	-	-	
Function	3	-	-	6	1	1	-	-	4	-	-	Assistance from another person for ADL, flexion
Health related quality of life	4	3	1	2	1	-	1	1	-	-	-	
Mental well-being	2	1	-	-	1	-	1	2	-	-	-	HOOS

*SF-36* 36-item short form health survey, *EQ-5D* EuroQol 5 Dimensions, *SF-12* 12-item short form health survey, *WOMAC* Western Ontario & McMaster Universities osteoarthritis index, *OHS* Oxford Hip score; *HHS* Harris Hip score, *HOOS* hip disability and osteoarthritis outcome score

^a^Reported in only one study

### Prognostic factors

#### Age

Eleven studies (31 %) reported that the outcome was significantly influenced by age (Additional file 2), of which five studies had low risk of bias. Two of these low risk of bias studies found a nonlinear relationship with age. Gordon et al. [38] found that outcomes were fairly unaffected by age until patients were in their late sixties, after which age had a negative effect on the EQ-5D. Judge et al. [40] found a small, not clinically relevant, effect of patients aged 50–60 reaching better postoperative Oxford Hip Scores (OHS). The three other studies found that older patients had smaller improvements or worse outcomes, but most differences were small [34, 36, 42]. Furthermore, the study of Cushnaghan et al. [34] was one of the few studies with a longer follow-up (~8 years) and a control group. Although a higher age predicted smaller changes in the SF-36 (Physical Function (PF)) in this study, this difference was also found in the control group suggesting that the effect is explained by ageing. Most of the high risk of bias studies also found that older patients had smaller improvements or worse outcomes, but that most differences were small [17, 32, 43–45]. Furthermore, Clement et al. [43] found that patients aged ≤80 years had a greater, but not clinically relevant improvement on the SF-12. Despite smaller improvements for older patients (>80 years), they were more satisfied after THA. Another study found that older patients (>75 years) had a higher revision rate than younger patients (65–75 years) [27]. Eight studies tested the association between age and outcomes such as SF-12 MCS, OHS (pain), post-operative complications, walking distance, LEFS, SF-36, WOMAC, EQ-5D and gait improvement, but did not find significant effects [16, 18, 21, 32, 41, 43–45]. One of these studies had low risk of bias [41].

#### Gender

Ten studies (29 %) reported associations between gender and outcomes in different directions. Three studies had low risk of bias. Cushnaghan et al. [34] reported that females had smaller improvements on the SF-36 (PF) scale. However, this was found in both cases and controls regardless of arthroplasty. Gandhi et al. [36] on the other hand, reported worse outcomes for males on the SF-36 (PF) and Gordon et al. [37] reported higher EQ-5D scores for males. Of the other high risk of bias studies, Greene et al. and Rolfson et al. [16, 23] found women were less satisfied. Heiberg et al. [17] found that males reached better scores of walking distance (on the 6-min walk test (6MWT)) (60.3 m more than women), which is a clinically relevant difference [46]. However, they did not use a control group and it may be that healthy male controls also reach better scores of walking distance compared to females. Furthermore, Katz et al. [27] found higher rates of revision in men than in women. Many studies investigated the association but did not find any significant associations of gender and various outcomes such as WOMAC, SF-36, pain, EQ VAS and gait improvement [16, 18, 21, 23, 31, 32, 36, 37, 41, 42, 45]. Four of these studies had low risk of bias [36, 37, 41, 42].

#### SES/education

Only three studies (9 %) reported an association between socioeconomic status or education and outcomes. None of these studies had low risk of bias. The studies reported more favorable outcomes following surgery in patients with a higher education [16, 18] or SES [47]. Sarasqueta et al. [21] did not find an association between education level and WOMAC.

#### Comorbidities

Comorbidities were associated with worse outcomes in 7 studies (20 %), of which four studies had low risk of bias [34, 36, 37, 40]. These low risk of bias studies found that patients with comorbidities had worse outcomes. However, the size of the effects varied from having a small effect for patients with comorbidities on the OHS [40] to a large effect for patients with diabetes on the SF-36 [34]. Gandhi et al. [36] found that patients with comorbidities scored worse on the WOMAC and the SF-36. Another low risk of bias study found that a higher Charnley comorbidity class was associated with worse outcomes on the EQ-5D [37]. The same results were also found in two high risk of bias studies [16, 23]. In addition, Judge et al. [48] found an association between number of painful joint sites and worse outcomes on the SF-36. However, six studies did not find significant associations between different comorbidities and outcomes such as SF-36, revision, chronic hip pain and WOMAC [21, 27, 30, 34, 41, 48]. Two of these studies had low risk of bias [34, 41].

#### Body mass index

Five studies (14 %) reported an association between BMI and postoperative outcomes. Two of these studies had low risk of bias [35, 40] where the study of Davis et al. [35] reported the largest effect with morbidly obese patients (BMI ≥35 kg/m^2^) having a 4.42 times higher dislocation rate than those with BMI <25 kg/m^2^. The authors also found associations between higher BMI and more superficial infections, poorer HHS and lower SF-36 postoperative scores [35]. Judge et al. [40] reported that patients with higher BMI had smaller absolute improvement on the OHS. However, regardless of their BMI, patients achieved substantial improvement in the OHS which outweighs the small absolute difference in attained OHS. The same was found in a high risk of bias study [19]. Other high risk of bias studies found that overweight and obesity were associated with a 3.7 fold increased risk of implant dislocation [25], and with lower SF-36 postoperative scores [49]. Furthermore, eight studies did not find an association with BMI and different outcomes, such as 6MWT, LEFS, WOMAC, SF-36 and chronic hip pain [21, 30, 34, 36, 41, 42, 44, 48]. Four of these studies had low risk of bias [34, 36, 41, 42].

#### Radiological OA severity

Six studies (17 %) reported significant associations between radiological OA severity and outcomes. Only one study had low risk of bias [34]. This study found that changes in physical functioning were markedly better in those with worse preoperative radiological OA grades. This was also found in two other high risk of bias studies [20, 48]. However, these studies focused on changes and not on final outcomes. Patients with lower baseline scores are more likely to improve, but the question is whether they reach the same postoperative levels. Another high risk of bias study found that patients with less severe radiological change had better postoperative outcomes [18]. Furthermore, Tanaka et al. [45] showed that a worse radiological OA stage predicted worse gait improvements after surgery. On the other hand, Meding et al. [22] found that patients with a greater degree of preoperative cartilage space loss had less hip pain 1 year after surgery, but no association was found at 3 years after surgery. Nilsdotter et al. [31] found that patients with severe preoperative radiological OA did not differ in postoperative outcome compared with patients with only moderate preoperative radiological OA.

#### Patient expectations

Two included high risk of bias studies (6 %) reported an association between patient expectations and outcomes. Bethge et al. [14] found that patients who expected an enduring illness and did not expect treatment to be helpful had worse postoperative scores on the HHS. Judge et al. [18] showed that patients with high expectations were more likely to improve on the WOMAC scale.

#### Pain

Six studies (17 %) reported an effect of preoperative pain on outcomes. The results were conflicting. Two studies that had low risk of bias showed that pain was related to worse outcomes. Nilsdotter et al. [42] reported that a higher degree of pain predicted worse function at 3.6 years after surgery. McHugh et al. [41] found that worse pain at baseline was negatively associated with improvement. In other high risk of bias studies, patients with the worst pre-operative WOMAC pain scores and SF-36 (Bodily Pain) also performed worse at 1 year postoperatively [32]. On the other hand, Judge et al. [18] found that patients with worse baseline pain had a greater improvement post-surgery on pain. Haverkamp et al. [28] showed that more preoperative pain at rest or at night resulted in more improvement on the WOMAC and VAS pain scale, but the patients maintained at a lower level at final follow up. Furthermore, Street et al. [33] looked at different pain areas and found that patients with knee pain showed less improvement (on HHS, WOMAC and SF-36) than those with hip or thigh pain. Röder et al. [24] concluded that pain relief was independent of the preoperative pain level. No significant associations were found in 5 other studies with outcomes such as pain, WOMAC and satisfaction [21, 24, 28, 30, 39]. One of these studies had low risk of bias [39].

#### Function

Several questionnaires were used to assess preoperative function and associations were found in 13 studies (37 %). Two of these studies had low risk of bias [34, 39]. One of these studies showed that patients with a worse preoperative function had a greater improvement [34], which was also found in other studies [18, 43, 48]. The other low risk of bias study showed that although patients with worse preoperative function had a greater improvement, they did not achieve the postoperative level of those with higher preoperative function [39]. This was also confirmed in other high risk of bias studies [17, 24, 29, 32, 40, 44]. In most studies these observed differences were regarded as clinically relevant by the authors. Four studies did not find associations between function and various outcomes such as 6MWT, LEFS, ROM, deformity, HHS, SF-36 and gait improvement [17, 29, 44, 45]. None of these studies had low risk of bias.

#### Health related quality of life

Ten studies (29 %) reported significant associations between preoperative health related quality of life (HRQoL) and postoperative outcomes, three of these studies had low risk of bias [34, 40, 42]. In these low risk of bias studies, better preoperative quality of life was associated with better postoperative scores. Judge et al. [40] reported a small but statistically significant effect on the OHS. Nilsdotter [42] found an association with worse WOMAC scores. Cushnaghan [34] found that patients with a higher SF-36 score had less improvement postoperatively. This was also found in a high risk of bias study by Gordon et al. [38], in which the authors stated that patients with low preoperative scores had the highest gain, although they did not reach the same absolute levels as patients with high preoperative scores. No associations were found in eight studies that tested associations of different HRQoL scores on outcomes, such as WOMAC, pain, satisfaction, EQ-5D, SF-36 and WOMAC [14, 16, 18, 21, 23, 32, 36, 42]. Two of these studies had low risk of bias [36, 42].

#### Mental well-being

Five studies (14 %) reported that mental well-being, such as anxiety and depressive symptoms, was associated with postoperative outcomes. Two of these studies had low risk of bias and found that worse mental well-being was associated low OHS [40] and less change in SF-36 PCS [41]. The three other high risk of bias studies also found that worse mental well-being was associated with various worse outcomes, such as pain relief, EQ-5D, satisfaction, SF-36 and Hip disability and Osteoarthritis Outcome Score (HOOS) [15, 23, 49].

## Discussion

We know that THA improves clinical and functional outcomes in most patients, and for some more than others. We also know that some patients achieve better postoperative levels of these outcomes than other patients. Hence it is relevant to assess which variables predict the outcome and the extent of improvement after THA. Therefore, we performed a systematic review in which multiple preoperative factors were included. Our review shows that the results on which predictors affect specific outcomes after THA were not consistent, even when looking only at low risk of bias studies. Some predictors were examined in many studies, but the results were conflicting as to whether an association was found (e.g. for age, comorbidity, pain and preoperative health related quality of life). Sometimes the associations could even go in different directions such as for gender. Other predictors were only reported in a few studies, such as SES/education, patient expectations, and mental well-being. Consistent and clinically relevant effects on postoperative outcomes were only found for preoperative radiological OA severity and preoperative function. However, only one study that assessed radiological OA severity and two studies that assessed preoperative function had low risk of bias. Overall, even though greater improvements were found in patients with more severe radiological OA and lower function baseline scores, these patients did not reach the same postoperative levels in functioning as patients with less severe OA or higher baseline function scores. Moreover, these associations were not found in all studies [17, 29, 31, 44, 45] and these studies had a high risk of bias.

Even though BMI is often considered as a relevant predictor of postoperative outcome, our review shows that only 5 out of the 13 studies (2 low risk of bias studies) reported a significant association between BMI and outcomes. Furthermore, complication rates after surgery were higher for patients with a higher BMI, but the patient reported outcomes did not show clinically relevant differences depending on BMI in both low and high risk of bias studies. This may be explained partly because we focused on long term follow-up (≥1 year) and did not investigate short term complications, which more often occur in patients with a higher BMI. Patients achieved substantial improvement in the patient reported outcomes regardless of their BMI [19, 35] so that patients should not be withheld from surgery only because of their high BMI. Furthermore, age was a major confounder in many studies, as with increasing age people tend to be for example less physically active and may have comorbidities as part of a physiological aging process which will bias the observed associations between other predictors and outcomes. As a result, some studies may have found smaller improvement in elderly people. However, it may be possible that elderly people are satisfied with a small improvement since their lifestyle may be less active as well. Since only one study compared the outcomes with a control group (without THA), it is difficult to conclude whether differences are based on the “prognostic” factor or that it is just the natural course of life.

An earlier systematic review on preoperative predictors on outcomes in THA [50] included studies until 2005. They concluded that THA resulted in pain relief, improved physical function and enhanced health-related quality of life regardless of patients’ characteristics, type of operation or type of prosthesis. The only factor affecting patient outcomes was patients’ poor preoperative function. Furthermore, the authors did not perform a risk of bias assessment. Most studies included in the present review were published after 2005 (31 of the 35). Still, we found similar results even when focusing on low risk of bias studies only. Furthermore, two reviews focused on patients’ characteristics. Santaguida et al. [11] found in their systematic review that age and gender were associated with risk of revision and mortality after total hip and knee arthroplasty and that age was associated with function. However, they found that all patients benefited from total joint arthroplasty regardless of their age and gender. Waheeb et al. [10] also showed that high variability and conflicting findings were reported on the effect of age, gender and BMI on patient reported outcomes. While these reviews focused on patients’ characteristics, our review adds how other factors such as radiological OA severity, preoperative quality of life and preoperative function affect postoperative outcomes.

Studies in our systematic review were heterogeneous and differed in follow-up time (beyond one year), prognostic factors and outcomes, which may explain the conflicting findings and make it difficult to compare studies. It also shows that there is no consensus in which outcomes should be used to assess the impact of surgery and which prognostic factors should be considered. Differences in reported associations may be partly explained by differences in the measurement of these predictors and outcomes (e.g. function is measured with HOOS, WOMAC, OHS etc.). The majority of the included studies assessed outcomes through self-reported questionnaires, which may bias results due to response shift [51]. Patients may report changes over time due to changes in their internal standards, values, or conceptualization of health related quality of life [51] so that it seems as if scores change, but this may not be reflected in objective measurements. In addition, radiological OA severity may vary due to inter- and intra-observer variability. Therefore, more uniformity is needed regarding types of measurements and questionnaires. Furthermore, some studies focused on improvements while other studies focused on the final outcome, so that regression to the mean should be taken into account.

Loss to follow-up was a problem in 18 studies, which is likely to bias the associations found. For instance, patients who are less satisfied or have poor outcome after a THA are less likely to further participate in a study and therefore be lost to follow-up. Hence, satisfied patients with good outcomes may be over-represented [52]. Another problem may be reporting bias. Although some authors described both significant associations and non-significant associations within a study, it is likely that the same associations were investigated by others, but not reported if results were non-significant. Since most studies examining these topics were observational studies, outcome reporting bias is possible as primary outcomes of observational studies are not documented in a trial register as for randomized controlled trials. Furthermore, predictors and outcomes were measured with questionnaires covering multiple domains. For example the SF-36 has eight domains and two summary scores (MCS and PCS). Studies using these questionnaires often did not correct for multiple testing so that it is possible that some associations were in fact chance findings (5 %). Also some of the studies included overlapping cohorts, but most often did assess different prognostic variables on different outcomes. A strength of this review is the strict inclusion criteria concerning patients with primary OA who underwent a THA. This made the populations in the selected studies better comparable. This also led to exclusion of many studies that analyzed THA and TKA as one group or included other patient groups. Since THA and TKA are two different surgeries including these studies would have made results even more heterogeneous.

## Conclusion

In this systematic review we synthesized information about multiple preoperative factors and their relation with postoperative outcomes. However, there is too little high quality evidence to draw firm conclusions on prognostics factors for specific outcomes after THA. Overall, preoperative function and radiological OA were predictors with the most consistent findings in studies with low risk of bias. Worse preoperative function and more severe radiological OA were associated with larger postoperative improvement. However, these patients did not reach the level of postoperative functioning as patients with better preoperative function or less severe radiological OA. The present mapping of current evidence on the relationship between patient related factors and outcomes provides better information compared to individual studies and may help to set patient expectations before surgery.

### Implications for future research

Insight into preoperative patient related factors and their relation with postoperative outcomes brings us a step closer to the determination of the optimal timing of THA. Procedures should not be performed too early, as the lifespan of a prosthesis is limited, and revision arthroplasty is less successful than primary TKA or THA [53]. A surgeon could possibly decide to postpone a THA by first optimizing preoperative function using different non-surgical treatments, if patients would then reach the same or better postoperative functional levels. Therefore, further research is needed to determine optimal preoperative (range of) cutoff points to recommend implant surgery, using a patients’ lifetime perspective and our results on which preoperative factors determine the outcomes after THA. In addition, as we focused on patient related factors only, there are also many other factors that might influence the outcome, such as type of prosthesis (e.g. type of stem, head size, cemented/uncemented), experience of the surgeon or hospital type. These factors should also be taken into account when determining the optimal timing of surgery.

### Ethics approval and consent to participate

Not applicable.

### Consent for publication

Not applicable.

### Availability of data and materials

The data supporting the conclusions of this article are included within the article and its additional files.

## Additional files

Additional file 1: Search Strategy. (DOCX 30 kb) Additional file 2: Reported predictors for outcomes. (DOCX 73 kb)

## Abbreviations

6MWT: 6 minute walk test
ADL: activities of daily living
ASA: American society of anesthesiologists
ATD: Articulotrochanteric distance
BIPQ: the brief illness perception questionnaire
BMI: body mass index
DEPCAT: deprivation categories
EQ-5D: EuroQol 5 dimensions
ESSI: the ENRICHD social support instrument
HADS: hospital anxiety and depression scale
HHS: Harris Hip score
HOOS: hip disability and osteoarthritis outcome score
K&L grade: Kellgren-Lawrence
LEFS: lower extremity functional scale
LLD: leg length discrepancy
OA: osteoarthritis
OHS: Oxford hip score
QOL: quality of life
ROM: range of motion
SF-12: 12-item short form health survey
SF-36: 36-item Short Form Health Survey (subscales PF: Physical Functioning; RP: Physical Role; BP: Bodily Pain; GH; General Health; VT: Vitality; SF: Social Functioning; RE: Role-Emotional; MH: Mental Health; MCS: Mental Component Summary score; PCS: Physical Component Summary score)
THA: total hip arthroplasty
THR: total hip replacement
VAS: visual analogue scale
WOMAC: Western Ontario & McMaster Universities osteoarthritis index
