# Supplementary material for: Preoperative predictors for outcomes after total hip replacement in patients with osteoarthritis: a systematic review
Source: BMC Musculoskelet Disord. 2016 May 17;17:212. doi: 10.1186/s12891-016-1070-3 (PMC4869370; doi:10.1186/s12891-016-1070-3)
Supplement: Additional file 1: — Search Strategy. (DOCX 30 kb) [file 12891_2016_1070_MOESM1_ESM.docx]

**Additional file 1. Search Strategy**

| **Databases** | Search Strategy | Number of references | Number of unique references |
| --- | --- | --- | --- |
|  |  |  |  |
| PubMed | ("Osteoarthritis"[mesh] OR "Osteoarthritis"[tw] OR osteoarthrit*[tw] OR "osteoarthrosis"[tw] OR "osteoarthroses"[tw] OR "Degenerative Arthritis"[all fields] OR "coxarthrosis"[tw] OR "gonarthrosis"[tw] OR "oa"[tw]) AND ((("Arthroplasty"[majr] OR "Joint Prosthesis"[majr] OR "Prostheses and Implants"[majr] OR arthroplast*[tiab] OR joint prosthe*[tiab] OR prosthe*[tiab]) AND ("Hip"[majr] OR "Hip Joint"[majr] OR hip[tiab] OR hips[tiab])) OR (("Arthroplasty, Replacement, Hip"[majr] OR "Hip Prosthesis"[majr] OR "hip prosthesis"[tiab] OR "hip prostheses"[tiab] OR "hip replacement"[tiab] OR "hip replacements"[tiab] OR "hip arthroplasty"[tiab] OR "hip arthroplasties"[tiab] OR (("tha"[tiab] OR "thr"[tiab] OR "thas"[tiab] OR "thrs"[tiab]) AND (hip[tiab] OR hips[tiab] OR replace*[tiab] OR replacement[tiab] OR replacing[tiab] OR replaced[tiab] OR arthroplast*[tiab] OR arthroplasty[tiab] OR arthroplastic[tiab] OR prosthe*[tiab] OR prosthesis[tiab] OR prostheses[tiab] OR prosthetic[tiab] OR endoprosthe*[tiab] OR implant*[tiab] OR implant[tiab] OR implants[tiab] OR implanted[tiab])) OR ((Hip[ti] OR Hips[ti] OR Hip*[ti]) AND (replace*[ti] OR replacement[ti] OR replacing[ti] OR replaced[ti] OR arthroplast*[ti] OR arthroplasty[ti] OR arthroplastic[ti] OR prosthe*[ti] OR prosthesis[ti] OR prostheses[ti] OR prosthetic[ti] OR endoprosthe*[ti] OR implant*[ti] OR implant[ti] OR implants[ti] OR implanted[ti]))))) AND ("determinant"[tw] OR "determinants"[tw] OR "predictor"[tw] OR "predictors"[tw] OR "predictive"[tw] OR "Predictive Value of Tests"[Mesh] OR "factor"[tw] OR "factors"[tw] OR "Epidemiologic Factors"[Mesh] OR "Risk Factors"[mesh] OR "associated"[tw] OR "association"[tw] OR "association"[mesh] OR "cause"[tw] OR "causal"[tw] OR "Causality"[Mesh] OR "causality"[tw] OR "attribute"[tw] OR "attributes"[tw] OR "Psychometrics"[Mesh] OR "psychometrics"[tw] OR psychometric*[tw] OR "prognostic"[tw] OR "Prognosis"[Mesh:noexp] OR "Prognosis"[tw] OR "timing"[tw] OR "Time Factors"[mesh] OR "non-surgical"[tw] OR "nonsurgical"[tw] OR "conservative"[tw]) AND ("postoperative"[tw] OR "post-operative"[tw] OR "Postoperative Period"[Mesh] OR "Postoperative Complications"[Mesh] OR "Postoperative Care"[Mesh] OR "postsurgical"[tw] OR "postsurgery"[tw] OR "post-surgical"[tw] OR "post-surgery"[tw]) AND ("QOL"[tw] OR "Quality of Life"[mesh] OR "quality of life"[tw] OR "Pain"[mesh] OR "pain"[tw] OR "Pain Measurement"[mesh] OR "function"[tw] OR functional[tw] OR "Range of Motion, Articular"[Mesh] OR "Mobility Limitation"[mesh] OR "Recovery of Function"[Mesh] OR "Musculoskeletal Physiological Phenomena"[Mesh] OR "adverse"[tw] OR "adverse effects" [Subheading] OR "Prosthesis Failure"[mesh] OR "mortality"[tw] OR "Mortality"[Mesh] OR "mortality"[Subheading] OR "revision"[tw] OR "Reoperation"[Mesh] OR "Reoperation"[tw] OR "outcome"[tw] OR "outcomes"[tw] OR "Outcome and Process Assessment (Health Care)"[Mesh] OR "clinical"[tw]) NOT ("Animals"[mesh] NOT "Humans"[mesh])  **Three additional strategies for PubMed only:**  **1.**  (hip[ti] OR hips[ti] OR tha[ti] OR thr[ti] OR (("total joint"[ti]) AND (hip[tw] OR hips[tw]))) AND (((("Arthroplasty"[majr] OR "Joint Prosthesis"[majr] OR "Prostheses and Implants"[majr] OR arthroplast*[ti] OR joint prosthe*[ti] OR prosthe*[ti]) AND ("Hip"[majr] OR "Hip Joint"[majr] OR hip[ti] OR hips[ti])) OR (("Arthroplasty, Replacement, Hip"[majr] OR "Hip Prosthesis"[majr] OR "hip prosthesis"[ti] OR "hip prostheses"[ti] OR "hip replacement"[ti] OR "hip replacements"[ti] OR "hip arthroplasty"[ti] OR "hip arthroplasties"[ti] OR (("tha"[ti] OR "thr"[ti] OR "thas"[ti] OR "thrs"[ti]) AND (hip[ti] OR hips[ti] OR replace*[ti] OR replacement[ti] OR replacing[ti] OR replaced[ti] OR arthroplast*[ti] OR arthroplasty[ti] OR arthroplastic[ti] OR prosthe*[ti] OR prosthesis[ti] OR prostheses[ti] OR prosthetic[ti] OR endoprosthe*[ti] OR implant*[ti] OR implant[ti] OR implants[ti] OR implanted[ti])) OR ((Hip[ti] OR Hips[ti] OR Hip*[ti]) AND (replace*[ti] OR replacement[ti] OR replacing[ti] OR replaced[ti] OR arthroplast*[ti] OR arthroplasty[ti] OR arthroplastic[ti] OR prosthe*[ti] OR prosthesis[ti] OR prostheses[ti] OR prosthetic[ti] OR endoprosthe*[ti] OR implant*[ti] OR implant[ti] OR implants[ti] OR implanted[ti]))))) AND ("determinant"[ti] OR "determinants"[ti] OR "predictor"[ti] OR "predictors"[ti] OR "predictive"[ti] OR "Predictive Value of Tests"[majr] OR "factor"[ti] OR "factors"[ti] OR "Epidemiologic Factors"[majr] OR "Risk Factors"[majr] OR "associated"[ti] OR "association"[ti] OR "association"[majr] OR "cause"[ti] OR "causal"[ti] OR "Causality"[majr] OR "causality"[ti] OR "attribute"[ti] OR "attributes"[ti] OR "Psychometrics"[majr] OR "psychometrics"[ti] OR psychometric*[ti] OR "prognostic"[ti] OR "Prognosis"[majr:noexp] OR "Prognosis"[ti] OR "timing"[ti] OR "Time Factors"[majr] OR "non-surgical"[ti] OR "nonsurgical"[ti] OR "conservative"[ti]) AND ("postoperative"[ti] OR "post-operative"[ti] OR "Postoperative Period"[majr] OR "Postoperative Complications"[majr] OR "Postoperative Care"[majr] OR "postsurgical"[ti] OR "postsurgery"[ti] OR "post-surgical"[ti] OR "post-surgery"[ti] OR "after"[ti]) AND ("QOL"[ti] OR "Quality of Life"[majr] OR "quality of life"[ti] OR "Pain"[majr] OR "pain"[ti] OR "Pain Measurement"[majr] OR "function"[ti] OR functional[ti] OR "Range of Motion, Articular"[majr] OR "Mobility Limitation"[majr] OR "Recovery of Function"[majr] OR "Musculoskeletal Physiological Phenomena"[majr] OR "adverse"[ti] OR "adverse effects" [Subheading] OR "Prosthesis Failure"[majr] OR "mortality"[ti] OR "Mortality"[majr] OR "mortality"[Subheading] OR "revision"[ti] OR "Reoperation"[majr] OR "Reoperation"[ti] OR "outcome"[ti] OR "outcomes"[ti] OR "Outcome and Process Assessment (Health Care)"[majr] OR "clinical"[ti]) NOT ("Animals"[majr] NOT "Humans"[majr]))  **2.**  (((("Arthroplasty"[majr] OR "Joint Prosthesis"[majr] OR "Prostheses and Implants"[majr] OR arthroplast*[tiab] OR joint prosthe*[tiab] OR prosthe*[tiab]) AND ("Hip"[majr] OR "Hip Joint"[majr] OR hip[tiab] OR hips[tiab])) OR (("Arthroplasty, Replacement, Hip"[majr] OR "Hip Prosthesis"[majr] OR "hip prosthesis"[tiab] OR "hip prostheses"[tiab] OR "hip replacement"[tiab] OR "hip replacements"[tiab] OR "hip arthroplasty"[tiab] OR "hip arthroplasties"[tiab] OR (("tha"[tiab] OR "thr"[tiab] OR "thas"[tiab] OR "thrs"[tiab]) AND (hip[tiab] OR hips[tiab] OR replace*[tiab] OR replacement[tiab] OR replacing[tiab] OR replaced[tiab] OR arthroplast*[tiab] OR arthroplasty[tiab] OR arthroplastic[tiab] OR prosthe*[tiab] OR prosthesis[tiab] OR prostheses[tiab] OR prosthetic[tiab] OR endoprosthe*[tiab] OR implant*[tiab] OR implant[tiab] OR implants[tiab] OR implanted[tiab])) OR ((Hip[ti] OR Hips[ti] OR Hip*[ti]) AND (replace*[ti] OR replacement[ti] OR replacing[ti] OR replaced[ti] OR arthroplast*[ti] OR arthroplasty[ti] OR arthroplastic[ti] OR prosthe*[ti] OR prosthesis[ti] OR prostheses[ti] OR prosthetic[ti] OR endoprosthe*[ti] OR implant*[ti] OR implant[ti] OR implants[ti] OR implanted[ti]))))) AND ("determinant"[tw] OR "determinants"[tw] OR "predictor"[tw] OR "predictors"[tw] OR "predictive"[tw] OR "Predictive Value of Tests"[Mesh] OR "factor"[tw] OR "factors"[tw] OR "Epidemiologic Factors"[Mesh] OR "Risk Factors"[mesh] OR "associated"[tw] OR "association"[tw] OR "association"[mesh] OR "cause"[tw] OR "causal"[tw] OR "Causality"[Mesh] OR "causality"[tw] OR "attribute"[tw] OR "attributes"[tw] OR "Psychometrics"[Mesh] OR "psychometrics"[tw] OR psychometric*[tw] OR "prognostic"[tw] OR "Prognosis"[Mesh:noexp] OR "Prognosis"[tw] OR "timing"[tw] OR "Time Factors"[mesh] OR "non-surgical"[tw] OR "nonsurgical"[tw] OR "conservative"[tw]) AND ("postoperative"[tw] OR "post-operative"[tw] OR "Postoperative Period"[Mesh] OR "Postoperative Complications"[Mesh] OR "Postoperative Care"[Mesh] OR "postsurgical"[tw] OR "postsurgery"[tw] OR "post-surgical"[tw] OR "post-surgery"[tw] OR "after"[tiab]) AND ("Patient Satisfaction"[majr] OR "Activities of Daily Living"[majr]) NOT ("Animals"[mesh] NOT "Humans"[mesh]))  **3.**  (("Osteoarthritis"[mesh] OR "Osteoarthritis"[tw] OR osteoarthrit*[tw] OR "osteoarthrosis"[tw] OR "osteoarthroses"[tw] OR "Degenerative Arthritis"[all fields] OR "coxarthrosis"[tw] OR "gonarthrosis"[tw] OR "oa"[tw]) AND ((("Arthroplasty"[majr] OR "Joint Prosthesis"[majr] OR "Prostheses and Implants"[majr] OR arthroplast*[tiab] OR joint prosthe*[tiab] OR prosthe*[tiab]) AND ("Hip"[majr] OR "Hip Joint"[majr] OR hip[tiab] OR hips[tiab])) OR (("Arthroplasty, Replacement, Hip"[majr] OR "Hip Prosthesis"[majr] OR "hip prosthesis"[tiab] OR "hip prostheses"[tiab] OR "hip replacement"[tiab] OR "hip replacements"[tiab] OR "hip arthroplasty"[tiab] OR "hip arthroplasties"[tiab] OR (("tha"[tiab] OR "thr"[tiab] OR "thas"[tiab] OR "thrs"[tiab]) AND (hip[tiab] OR hips[tiab] OR replace*[tiab] OR replacement[tiab] OR replacing[tiab] OR replaced[tiab] OR arthroplast*[tiab] OR arthroplasty[tiab] OR arthroplastic[tiab] OR prosthe*[tiab] OR prosthesis[tiab] OR prostheses[tiab] OR prosthetic[tiab] OR endoprosthe*[tiab] OR implant*[tiab] OR implant[tiab] OR implants[tiab] OR implanted[tiab])) OR ((Hip[ti] OR Hips[ti] OR Hip*[ti]) AND (replace*[ti] OR replacement[ti] OR replacing[ti] OR replaced[ti] OR arthroplast*[ti] OR arthroplasty[ti] OR arthroplastic[ti] OR prosthe*[ti] OR prosthesis[ti] OR prostheses[ti] OR prosthetic[ti] OR endoprosthe*[ti] OR implant*[ti] OR implant[ti] OR implants[ti] OR implanted[ti]))))) AND ("determinant"[tw] OR "determinants"[tw] OR "predictor"[tw] OR "predictors"[tw] OR "predictive"[tw] OR "Predictive Value of Tests"[Mesh] OR "factor"[tw] OR "factors"[tw] OR "Epidemiologic Factors"[Mesh] OR "Risk Factors"[mesh] OR "associated"[tw] OR "association"[tw] OR "association"[mesh] OR "cause"[tw] OR "causal"[tw] OR "Causality"[Mesh] OR "causality"[tw] OR "attribute"[tw] OR "attributes"[tw] OR "Psychometrics"[Mesh] OR "psychometrics"[tw] OR psychometric*[tw] OR "prognostic"[tw] OR "Prognosis"[Mesh:noexp] OR "Prognosis"[tw] OR "timing"[tw] OR "Time Factors"[mesh] OR "non-surgical"[tw] OR "nonsurgical"[tw] OR "conservative"[tw]) AND ("postoperative"[tw] OR "post-operative"[tw] OR "Postoperative Period"[Mesh] OR "Postoperative Complications"[Mesh] OR "Postoperative Care"[Mesh] OR "postsurgical"[tw] OR "postsurgery"[tw] OR "post-surgical"[tw] OR "post-surgery"[tw] OR "after"[tiab]) AND ("QOL"[tw] OR "Quality of Life"[mesh] OR "quality of life"[tw] OR "Pain"[mesh] OR "pain"[tw] OR "Pain Measurement"[mesh] OR "function"[tw] OR functional[tw] OR "Range of Motion, Articular"[Mesh] OR "Mobility Limitation"[mesh] OR "Recovery of Function"[Mesh] OR "Musculoskeletal Physiological Phenomena"[Mesh] OR "adverse"[tw] OR "adverse effects" [Subheading] OR "Prosthesis Failure"[mesh] OR "mortality"[tw] OR "Mortality"[Mesh] OR "mortality"[Subheading] OR "revision"[tw] OR "Reoperation"[Mesh] OR "Reoperation"[tw] OR "outcome"[tw] OR "outcomes"[tw] OR "Outcome and Process Assessment (Health Care)"[Mesh] OR "clinical"[tw]) NOT ("Animals"[mesh] NOT "Humans"[mesh])) | 2.040 | 2.040 |
|  |  |  |  |
| MEDLINE (OVID-version) | (exp Osteoarthritis/ OR "Osteoarthritis".mp OR osteoarthrit*.mp OR "osteoarthrosis".mp OR "osteoarthroses".mp OR "Degenerative Arthritis".mp OR "coxarthrosis".mp OR "gonarthrosis".mp OR "oa".mp) AND (((exp *Arthroplasty/ OR exp *Joint Prosthesis/ OR exp *"Prostheses and Implants"/ OR arthroplast*.ti,ab OR joint prosthe*.ti,ab OR prosthe*.ti,ab) AND (exp *Hip/ OR exp *Hip Joint/ OR hip.ti,ab OR hips.ti,ab)) OR ((exp *"Arthroplasty, Replacement, Hip"/ OR exp *Hip Prosthesis/ OR "hip prosthesis".ti,ab OR "hip prostheses".ti,ab OR "hip replacement".ti,ab OR "hip replacements".ti,ab OR "hip arthroplasty".ti,ab OR "hip arthroplasties".ti,ab OR (("tha".ti,ab OR "thr".ti,ab OR "thas".ti,ab OR "thrs".ti,ab) AND (hip.ti,ab OR hips.ti,ab OR replace*.ti,ab OR replacement.ti,ab OR replacing.ti,ab OR replaced.ti,ab OR arthroplast*.ti,ab OR arthroplasty.ti,ab OR arthroplastic.ti,ab OR prosthe*.ti,ab OR prosthesis.ti,ab OR prostheses.ti,ab OR prosthetic.ti,ab OR endoprosthe*.ti,ab OR implant*.ti,ab OR implant.ti,ab OR implants.ti,ab OR implanted.ti,ab)) OR ((Hip.ti OR Hips.ti OR Hip*.ti) AND (replace*.ti OR replacement.ti OR replacing.ti OR replaced.ti OR arthroplast*.ti OR arthroplasty.ti OR arthroplastic.ti OR prosthe*.ti OR prosthesis.ti OR prostheses.ti OR prosthetic.ti OR endoprosthe*.ti OR implant*.ti OR implant.ti OR implants.ti OR implanted.ti))))) AND ("determinant".mp OR "determinants".mp OR "predictor".mp OR "predictors".mp OR "predictive".mp OR exp "Predictive Value of Tests"/ OR "factor".mp OR "factors".mp OR exp "Epidemiologic Factors"/ OR exp "Risk Factors"/ OR "associated".mp OR "association".mp OR exp "association"/ OR "cause".mp OR "causal".mp OR exp Causality/ OR "causality".mp OR "attribute".mp OR "attributes".mp OR exp Psychometrics/ OR "psychometrics".mp OR psychometric*.mp OR "prognostic".mp OR Prognosis/ OR "Prognosis".mp OR "timing".mp OR exp Time Factors/ OR "non-surgical".mp OR "nonsurgical".mp OR "conservative".mp) AND ("postoperative".mp OR "post-operative".mp OR exp Postoperative Period/ OR exp Postoperative Complications/ OR exp Postoperative Care/ OR "postsurgical".mp OR "postsurgery".mp OR "post-surgical".mp OR "post-surgery".mp OR "after surgery".mp OR "after total hip".mp OR "after hip".mp OR "after tha".mp OR "after thr".mp) AND ("QOL".mp OR exp "Quality of Life"/ OR "quality of life".mp OR exp Pain/ OR "pain".mp OR exp Pain Measurement/ OR "function".mp OR functional.mp OR exp "Range of Motion, Articular"/ OR exp Mobility Limitation/ OR exp "Recovery of Function"/ OR exp "Musculoskeletal Physiological Phenomena"/ OR "adverse".mp OR "adverse effects".fs OR exp "Prosthesis Failure"/ OR "mortality".mp OR exp Mortality/ OR "mortality".fs OR "revision".mp OR exp Reoperation/ OR "Reoperation".mp OR "outcome".mp OR "outcomes".mp OR exp "Outcome and Process Assessment (Health Care)"/ OR "clinical".mp) NOT (exp Animals/ NOT exp Humans/) | 1.569 | 39 |
|  |  |  |  |
| Embase (OVID-version) | (exp *Osteoarthritis/ OR "Osteoarthritis".ti,ab OR osteoarthrit*.ti,ab OR "osteoarthrosis".ti,ab OR "osteoarthroses".ti,ab OR "Degenerative Arthritis".ti,ab OR "coxarthrosis".ti,ab OR "gonarthrosis".ti,ab) AND (((exp *Arthroplasty/ OR exp *Joint Prosthesis/ OR exp * "orthopedic prosthesis and orthosis"/ OR exp *"orthopedic prostheses, orthoses and implants"/ OR arthroplast*.ti OR joint prosthe*.ti,ab OR prosthe*.ti) AND (exp *Hip/ OR hip.ti OR hips.ti)) OR ((exp *hip arthroplasty/ OR exp *Hip Prosthesis/ OR "hip prosthesis".ti,ab OR "hip prostheses".ti,ab OR "hip replacement".ti,ab OR "hip replacements".ti,ab OR "hip arthroplasty".ti,ab OR "hip arthroplasties".ti,ab OR (("tha".ti,ab OR "thr".ti,ab OR "thas".ti,ab OR "thrs".ti,ab) AND (hip.ti,ab OR hips.ti,ab OR replace*.ti,ab OR replacement.ti,ab OR replacing.ti,ab OR replaced.ti,ab OR arthroplast*.ti,ab OR arthroplasty.ti,ab OR arthroplastic.ti,ab OR prosthe*.ti,ab OR prosthesis.ti,ab OR prostheses.ti,ab OR prosthetic.ti,ab OR endoprosthe*.ti,ab OR implant*.ti,ab OR implant.ti,ab OR implants.ti,ab OR implanted.ti,ab)) OR ((Hip.ti OR Hips.ti OR Hip*.ti) AND (replace*.ti OR replacement.ti OR replacing.ti OR replaced.ti OR arthroplast*.ti OR arthroplasty.ti OR arthroplastic.ti OR prosthe*.ti OR prosthesis.ti OR prostheses.ti OR prosthetic.ti OR endoprosthe*.ti OR implant*.ti OR implant.ti OR implants.ti OR implanted.ti))))) AND ("determinant".mp OR "determinants".mp OR "predictor".mp OR "predictors".mp OR "predictive".mp OR exp predictor variable/ OR exp " prediction and forecasting"/ OR "factor".mp OR "factors".mp OR exp Epidemiology/ OR exp "Risk Factor"/ OR "associated".mp OR "association".mp OR exp association/ OR exp disease association/ OR "cause".mp OR "causal".mp OR exp Causality/ OR "causality".mp OR "attribute".mp OR "attributes".mp OR exp Psychometry/ OR "psychometrics".mp OR psychometr*.mp OR "prognostic".mp OR Prognosis/ OR "Prognosis".mp OR "timing".mp OR Time/ OR "non-surgical".mp OR "nonsurgical".mp OR "conservative".mp OR exp conservative treatment/) AND ("postoperative".mp OR "post-operative".mp OR exp Postoperative Period/ OR exp Postoperative Complication/ OR exp Postoperative Care/ OR "postsurgical".mp OR "postsurgery".mp OR "post-surgical".mp OR "post-surgery".mp OR "after surgery".mp OR "after total hip".mp OR "after hip".mp) AND ("QOL".mp OR exp "Quality of Life"/ OR "quality of life".mp OR exp Pain/ OR "pain".mp OR exp Pain Assessment/ OR "function".mp OR functional.mp OR exp "Range of Motion"/ OR exp walking difficulty/ OR exp convalescence/ OR exp musculoskeletal function/ OR "adverse".mp OR adverse outcome/ OR exp "Prosthesis Failure"/ OR "mortality".mp OR exp Mortality/ OR "mortality".fs OR "revision".mp OR exp Reoperation/ OR "Reoperation".mp OR "outcome".mp OR "outcomes".mp OR exp treatment outcome/) NOT (exp Animals/ NOT exp Humans/) | 941 | 297 |
|  |  |  |  |
| Web of Science | (TS=(Osteoarthritis OR "Osteoarthritis" OR osteoarthrit* OR "osteoarthrosis" OR "osteoarthroses" OR "Degenerative Arthritis" OR "coxarthrosis" OR "gonarthrosis" OR "oa") AND TI=(((Arthroplasty OR Joint Prosthesis OR arthroplast* OR joint prosthe* OR prosthe*) AND (Hip OR hip OR hips)) OR ((hip arthroplasty OR Hip Prosthesis OR "hip prosthesis" OR "hip prostheses" OR "hip replacement" OR "hip replacements" OR "hip arthroplasty" OR "hip arthroplasties" OR (("tha" OR "thr" OR "thas" OR "thrs") AND (hip OR hips OR replace* OR replacement OR replacing OR replaced OR arthroplast* OR arthroplasty OR arthroplastic OR prosthe* OR prosthesis OR prostheses OR prosthetic OR endoprosthe* OR implant* OR implant OR implants OR implanted)) OR ((Hip OR Hips OR Hip*) AND (replace* OR replacement OR replacing OR replaced OR arthroplast* OR arthroplasty OR arthroplastic OR prosthe* OR prosthesis OR prostheses OR prosthetic OR endoprosthe* OR implant* OR implant OR implants OR implanted)))))) AND TS=(("determinant" OR "determinants" OR "predictor" OR "predictors" OR "predictive" OR predictor variable OR " prediction and forecasting" OR "factor" OR "factors" OR Epidemiology OR "Risk Factor" OR "associated" OR "association" OR association OR disease association OR "cause" OR "causal" OR Causality OR "causality" OR "attribute" OR "attributes" OR Psychometry OR "psychometrics" OR psychometr* OR "prognostic" OR Prognosis OR "Prognosis" OR "timing" OR Time OR "non-surgical" OR "nonsurgical" OR "conservative" OR conservative treatment) AND ("postoperative" OR "post-operative" OR Postoperative Period OR Postoperative Complication OR Postoperative Care OR "postsurgical" OR "postsurgery" OR "post-surgical" OR "post-surgery" OR "after surgery" OR "after total hip" OR "after hip") AND ("QOL" OR "Quality of Life" OR "quality of life" OR Pain OR "pain" OR Pain Assessment OR "function" OR functional OR "Range of Motion" OR walking difficulty OR convalescence OR musculoskeletal function OR "adverse" OR adverse outcome OR "Prosthesis Failure" OR "mortality" OR Mortality OR "mortality" OR "revision" OR Reoperation OR "Reoperation" OR "outcome" OR "outcomes")) | 391 | 131 |
|  |  |  |  |
| COCHRANE Library | ((Osteoarthritis OR "Osteoarthritis" OR osteoarthrit* OR "osteoarthrosis" OR "osteoarthroses" OR "Degenerative Arthritis" OR "coxarthrosis" OR "gonarthrosis" OR "oa") AND (((Arthroplasty OR Joint Prosthesis OR arthroplast* OR joint prosthe* OR prosthe*) AND (Hip OR hip OR hips)) OR ((hip arthroplasty OR Hip Prosthesis OR "hip prosthesis" OR "hip prostheses" OR "hip replacement" OR "hip replacements" OR "hip arthroplasty" OR "hip arthroplasties" OR (("tha" OR "thr" OR "thas" OR "thrs") AND (hip OR hips OR replace* OR replacement OR replacing OR replaced OR arthroplast* OR arthroplasty OR arthroplastic OR prosthe* OR prosthesis OR prostheses OR prosthetic OR endoprosthe* OR implant* OR implant OR implants OR implanted)) OR ((Hip OR Hips OR Hip*) AND (replace* OR replacement OR replacing OR replaced OR arthroplast* OR arthroplasty OR arthroplastic OR prosthe* OR prosthesis OR prostheses OR prosthetic OR endoprosthe* OR implant* OR implant OR implants OR implanted)))))) AND (("determinant" OR "determinants" OR "predictor" OR "predictors" OR "predictive" OR predictor variable OR " prediction and forecasting" OR "factor" OR "factors" OR Epidemiology OR "Risk Factor" OR "associated" OR "association" OR association OR disease association OR "cause" OR "causal" OR Causality OR "causality" OR "attribute" OR "attributes" OR Psychometry OR "psychometrics" OR psychometr* OR "prognostic" OR Prognosis OR "Prognosis" OR "timing" OR Time OR "non-surgical" OR "nonsurgical" OR "conservative" OR conservative treatment) AND ("postoperative" OR "post-operative" OR Postoperative Period OR Postoperative Complication OR Postoperative Care OR "postsurgical" OR "postsurgery" OR "post-surgical" OR "post-surgery" OR "after surgery" OR "after total hip" OR "after hip") AND ("QOL" OR "Quality of Life" OR "quality of life" OR Pain OR "pain" OR Pain Assessment OR "function" OR functional OR "Range of Motion" OR walking difficulty OR convalescence OR musculoskeletal function OR "adverse" OR adverse outcome OR "Prosthesis Failure" OR "mortality" OR Mortality OR "mortality" OR "revision" OR Reoperation OR "Reoperation" OR "outcome" OR "outcomes" OR treatment outcome OR "clinical")) | 141 | 41 |
|  |  |  |  |
| CENTRAL | ((Osteoarthritis OR "Osteoarthritis" OR osteoarthrit* OR "osteoarthrosis" OR "osteoarthroses" OR "Degenerative Arthritis" OR "coxarthrosis" OR "gonarthrosis" OR "oa") AND (((Arthroplasty OR Joint Prosthesis OR arthroplast* OR joint prosthe* OR prosthe*) AND (Hip OR hip OR hips)) OR ((hip arthroplasty OR Hip Prosthesis OR "hip prosthesis" OR "hip prostheses" OR "hip replacement" OR "hip replacements" OR "hip arthroplasty" OR "hip arthroplasties" OR (("tha" OR "thr" OR "thas" OR "thrs") AND (hip OR hips OR replace* OR replacement OR replacing OR replaced OR arthroplast* OR arthroplasty OR arthroplastic OR prosthe* OR prosthesis OR prostheses OR prosthetic OR endoprosthe* OR implant* OR implant OR implants OR implanted)) OR ((Hip OR Hips OR Hip*) AND (replace* OR replacement OR replacing OR replaced OR arthroplast* OR arthroplasty OR arthroplastic OR prosthe* OR prosthesis OR prostheses OR prosthetic OR endoprosthe* OR implant* OR implant OR implants OR implanted)))))) AND (("determinant" OR "determinants" OR "predictor" OR "predictors" OR "predictive" OR predictor variable OR " prediction and forecasting" OR "factor" OR "factors" OR Epidemiology OR "Risk Factor" OR "associated" OR "association" OR association OR disease association OR "cause" OR "causal" OR Causality OR "causality" OR "attribute" OR "attributes" OR Psychometry OR "psychometrics" OR psychometr* OR "prognostic" OR Prognosis OR "Prognosis" OR "timing" OR Time OR "non-surgical" OR "nonsurgical" OR "conservative" OR conservative treatment) AND ("postoperative" OR "post-operative" OR Postoperative Period OR Postoperative Complication OR Postoperative Care OR "postsurgical" OR "postsurgery" OR "post-surgical" OR "post-surgery" OR "after surgery" OR "after total hip" OR "after hip") AND ("QOL" OR "Quality of Life" OR "quality of life" OR Pain OR "pain" OR Pain Assessment OR "function" OR functional OR "Range of Motion" OR walking difficulty OR convalescence OR musculoskeletal function OR "adverse" OR adverse outcome OR "Prosthesis Failure" OR "mortality" OR Mortality OR "mortality" OR "revision" OR Reoperation OR "Reoperation" OR "outcome" OR "outcomes" OR treatment outcome OR "clinical")) | 143 | 11 |
|  |  |  |  |
| CINAHL | ((Osteoarthritis OR "Osteoarthritis" OR osteoarthrit* OR "osteoarthrosis" OR "osteoarthroses" OR "Degenerative Arthritis" OR "coxarthrosis" OR "gonarthrosis" OR "oa") AND (((Arthroplasty OR Joint Prosthesis OR arthroplast* OR joint prosthe* OR prosthe*) AND (Hip OR hip OR hips)) OR ((hip arthroplasty OR Hip Prosthesis OR "hip prosthesis" OR "hip prostheses" OR "hip replacement" OR "hip replacements" OR "hip arthroplasty" OR "hip arthroplasties" OR (("tha" OR "thr" OR "thas" OR "thrs") AND (hip OR hips OR replace* OR replacement OR replacing OR replaced OR arthroplast* OR arthroplasty OR arthroplastic OR prosthe* OR prosthesis OR prostheses OR prosthetic OR endoprosthe* OR implant* OR implant OR implants OR implanted)) OR ((Hip OR Hips OR Hip*) AND (replace* OR replacement OR replacing OR replaced OR arthroplast* OR arthroplasty OR arthroplastic OR prosthe* OR prosthesis OR prostheses OR prosthetic OR endoprosthe* OR implant* OR implant OR implants OR implanted)))))) AND (("determinant" OR "determinants" OR "predictor" OR "predictors" OR "predictive" OR predictor variable OR " prediction and forecasting" OR "factor" OR "factors" OR Epidemiology OR "Risk Factor" OR "associated" OR "association" OR association OR disease association OR "cause" OR "causal" OR Causality OR "causality" OR "attribute" OR "attributes" OR Psychometry OR "psychometrics" OR psychometr* OR "prognostic" OR Prognosis OR "Prognosis" OR "timing" OR Time OR "non-surgical" OR "nonsurgical" OR "conservative" OR conservative treatment) AND ("postoperative" OR "post-operative" OR Postoperative Period OR Postoperative Complication OR Postoperative Care OR "postsurgical" OR "postsurgery" OR "post-surgical" OR "post-surgery" OR "after surgery" OR "after total hip" OR "after hip") AND ("QOL" OR "Quality of Life" OR "quality of life" OR Pain OR "pain" OR Pain Assessment OR "function" OR functional OR "Range of Motion" OR walking difficulty OR convalescence OR musculoskeletal function OR "adverse" OR adverse outcome OR "Prosthesis Failure" OR "mortality" OR Mortality OR "mortality" OR "revision" OR Reoperation OR "Reoperation" OR "outcome" OR "outcomes" OR treatment outcome OR "clinical")) | 157 | 36 |
| Total |  |  | 2.595 |
|  |  |  |  |
| **Trialregisters** |  |  |  |
|  |  |  |  |
| ClinicalTrials.gov  <http://clinicaltrials.gov/> | (predictor OR predictors OR predictive OR predicting OR prediction OR predictions OR determinant OR determinants) AND (outcome OR outcomes) AND hip AND osteoarthritis | 27 | 27 |
|  |  |  |  |
| Multi-register  <http://www.controlled-trials.com/mrct/> | (predictor OR predictors OR predictive OR predicting OR prediction OR predictions OR determinant OR determinants) AND (outcome OR outcomes) AND hip AND osteoarthritis | 19 | 19 |
|  |  |  |  |
