# Supplementary material for: Preoperative predictors for outcomes after total hip replacement in patients with osteoarthritis: a systematic review
Source: BMC Musculoskelet Disord. 2016 May 17;17:212. doi: 10.1186/s12891-016-1070-3 (PMC4869370; doi:10.1186/s12891-016-1070-3)
Supplement: Additional file 2: — Reported predictors for outcomes. (DOCX 73 kb) [file 12891_2016_1070_MOESM2_ESM.docx]

**Additional file 2. Reported predictors for outcomes**

| **First author, year** | **n** | **Females (%)** | **Age (mean)** | **Follow-up (years) §** | **Significant associations** | | |
| --- | --- | --- | --- | --- | --- | --- | --- |
|  |  |  |  |  | **Prognostic factor** | **Outcome** | **Direction*** |
| *Studies with low risk of bias across both domains* | | | | | | | |
| Cushnaghan, 2007[34] | 282 | 65 | 68 | ~8 | Higher SF-36 (PF) | Change SF-36 (PF) | ↓ |
|  |  |  |  |  | Female | Change SF-36 (PF) | ↓ |
|  |  |  |  |  | Higher age | Change SF-36 (PF) | ↓ |
|  |  |  |  |  | Diabetes | Change SF-36 (PF) | ↓ |
|  |  |  |  |  | Higher radiological grade | Change SF-36 (PF) | ↑ |
|  |  |  |  |  | Higher number of painful joint sites | Change SF-36 (PF) | ↓ |
| Davis, 2011[35] | 1,163 | 61 | 69 | 5 | Higher BMI | Dislocation | ↑ |
|  |  |  |  |  | Higher BMI | Superficial infection | ↑ |
|  |  |  |  |  | Higher BMI | HHS | ↓ |
|  |  |  |  |  | Higher BMI | SF-36 (except for the domains mental health and change in health) | ↓ |
| Fortin, 2002[39] | 84 | 59 | 65.7 | 2 | Higher WOMAC (physical function) | WOMAC (physical function) | ↑ |
|  |  |  |  |  | Higher WOMAC (physical function) | Assistance from another person for ADL | ↑ |
| Gandhi, 2010[36] | 636 | 54 | 63 | Mean 3.3 | Higher age | WOMAC | ↑ |
|  |  |  |  |  | Comorbidities | WOMAC | ↑ |
|  |  |  |  |  | Higher age | SF-36 (PF) | ↓ |
|  |  |  |  |  | Comorbidities | SF-36 (PF) | ↓ |
|  |  |  |  |  | Male | SF-36 (PF) | ↓ |
|  |  |  |  |  | Higher age | SF-36 (RP) | ↓ |
|  |  |  |  |  | Comorbidities | SF-36 (RP) | ↓ |
| Gordon, 2014[37] | 26,249 | 57 | 70 female 80 male | 1 | Female | EQ-5D | ↓ |
|  |  |  |  |  | Charnley class C | EQ-5D | ↓ |
| Gordon, 2014[38] | 27,245 | 57 | 67 | 1 | Higher age from 60 year | EQ-5D | ↓ |
|  |  |  |  |  | Higher age from 60 year | EQ VAS | ↓ |
|  |  |  |  |  | Lower EQ-5D | Improvement EQ-5D | ↑ |
|  |  |  |  |  | Lower EQ VAS | Improvement EQ VAS | ↑ |
| Judge, 2013[40] | 1,375 | 62 | 70.0 | each year up to 5 | Age 50-60 | OHS | ↑ |
|  |  |  |  |  | Higher BMI | OHS | ↓ |
|  |  |  |  |  | Comorbidities | OHS | ↓ |
|  |  |  |  |  | Lower SF-36 (Mental Health) | OHS (pain/ function) | ↓ |
|  |  |  |  |  | Lower OHS | OHS | ↓ |
| McHugh, 2013[41] | 206 | 57 | 66.3 | 1 | Higher ESSI (social support) | SF-36 (MCS) | ↑ |
|  |  |  |  |  | Previous joint replacement | Change in SF-36 (PCS) | ↓ |
|  |  |  |  |  | Taking NSAIDs or COX-2 inhibitors | Change in SF-36 (PCS) | ↑ |
|  |  |  |  |  | Higher HADS anxiety score | Change in SF-36 (PCS) | ↓ |
|  |  |  |  |  | Higher HADS depression score | Change in SF-36 (PCS) | ↓ |
|  |  |  |  |  | Higher WOMAC pain score | Change in SF-36 (PCS) | ↓ |
| Nilsdotter, 2003[42] | 198 | 54 | 71 | Mean 3.6 | Higher age | WOMAC function | ↑ |
|  |  |  |  |  | Lower SF-36 (BP) | WOMAC function | ↑ |
| *Studies with high/unclear risk of bias in at least one of the domains* | | | | | | | |
| Bethge, 2010[14] | 135 | 66 | 72 | 1 | Higher BIPQ (subscale Expecting an enduring illness) | HHS | ↓ |
|  |  |  |  |  | Higher BIPQ (subscales expectation treatment is helpful) | HHS | ↑ |
| Clement, 2011[43] | 163 ≥80y  376 <80y | 55  63 | 84  70 | 1 | Lower age | SF-12 PCS | ↑ |
|  |  |  |  |  | Higher age | Satisfaction | ↑ |
|  |  |  |  |  | Lower age | OHS (function) | ↑ |
|  |  |  |  |  | Higher age | Complications | ↑ |
| Clement, 2011[47] | 1,312 | 58 | 68 | 1 | Higher OHS | OHS | ↑ |
|  |  |  |  |  | Higher DEPCAT | OHS | ↓ |
|  |  |  |  |  | Higher DEPCAT | Satisfaction | ↓ |
| Duivenvoorden, 2013[15] | 140 | 64 | 68 | 1 | Anxiety symptoms | HOOS (subscales pain, ADL, sports, QOL) | ↓ |
|  |  |  |  |  | Anxiety symptoms | Satisfaction (overall, pain reduction, improvement ADL, improvement QOL) | ↓ |
|  |  |  |  |  | Depressive symptoms | HOOS (subscales pain symptoms, ADL, QOL) | ↓ |
|  |  |  |  |  | Depressive symptoms | Satisfaction (improvement QOL) | ↓ |
| Greene, 2014[16] | 11,464 | 64 | 54 | 1 | High education | EQ-5D index | ↑ |
|  |  |  |  |  | EQ-5D index | EQ-5D index | ↑ |
|  |  |  |  |  | Charnley class B or C | EQ-5D index | ↓ |
|  |  |  |  |  | Comorbidities | EQ-5D index | ↓ |
|  |  |  |  |  | Male | EQ-5D index | ↑ |
|  |  |  |  |  | High education | EQ VAS | ↑ |
|  |  |  |  |  | EQ-5D index | EQ VAS | ↑ |
|  |  |  |  |  | Second hip | EQ VAS | ↓ |
|  |  |  |  |  | Charnley class B or C | EQ VAS | ↓ |
|  |  |  |  |  | Comorbidities | EQ VAS | ↓ |
|  |  |  |  |  | High education | Pain VAS | ↓ |
|  |  |  |  |  | EQ-5D index | Pain VAS | ↓ |
|  |  |  |  |  | Widow/ married | Pain VAS | ↓ |
|  |  |  |  |  | Charnley class B or C | Pain VAS | ↑ |
|  |  |  |  |  | High education | Satisfaction VAS | ↓ |
|  |  |  |  |  | EQ-5D index | Satisfaction VAS | ↓ |
|  |  |  |  |  | Second hip | Satisfaction VAS | ↑ |
|  |  |  |  |  | Charnley class B or C | Satisfaction VAS | ↑ |
|  |  |  |  |  | Females | Satisfaction VAS | ↑ |
| Haverkamp, 2013[28] | 155 | 71† | 68.3† | Mean 2.3† | Pain at rest/ at night | Improvement WOMAC | ↑ |
|  |  |  |  |  | Pain at rest/ at night | Improvement VAS pain | ↑ |
| Heiberg, 2013[17] | 64 | 52 | 65 | 1 | Younger age | 6MWT | ↑ |
|  |  |  |  |  | Males | 6MWT | ↑ |
|  |  |  |  |  | Higher 6MWT | 6MWT | ↑ |
|  |  |  |  |  | ROM | 6MWT | ↑ |
| Ieiri, 2013[49] | 108 | 85 | 61.3 | 1 | Contralateral hip OA | SF-36 (PF, RP, MH, RE, BP, VT, GH, SF) | ↓ |
|  |  |  |  |  | Walking aids | SF-36 (PF, RP, MH, RE, BP, VT, GH, SF) | ↓ |
|  |  |  |  |  | Lower contralateral hip ROM | SF-36 (PF, RP, MH, RE, BP, VT, GH, SF) | ↓ |
|  |  |  |  |  | Lower affected hip ROM | SF-36 (PF, RP, MH, RE, BP, VT, GH, SF) | ↓ |
|  |  |  |  | 3 | Higher age | SF-36 (PF, RP) | ↓ |
|  |  |  |  |  | Walking aids | SF-36 (PF, RP) | ↓ |
|  |  |  |  |  | Higher BMI | SF-36 (PF, RP) | ↓ |
|  |  |  |  |  | Lower contralateral hip ROM | SF-36 (PF, RP) | ↓ |
|  |  |  |  |  | Higher pre SF-36 mental health | SF-36 (MH, RP, GH, SF, VT, RE, BP) | ↑ |
|  |  |  |  |  | Not living alone | SF-36 (MH, RP, GH, SF, VT, RE, BP) | ↑ |
|  |  |  |  |  | Not working | SF-36 (MH, RP, GH, SF, VT, RE, BP) | ↑ |
| Johansson, 2010[29] | 75 | 48 | 67 | 2 | HHS poor (vs good) | HHS | ↓ |
|  |  |  |  |  | HHS poor (vs good) | WOMAC | ↓ |
|  |  |  |  |  | HHS poor | SF-36 | ↓ |
| Judge, 2014[19] | 4,413 | 62 | 68.5 | 1 | Higher BMI | OHS | ↓ |
| Judge, 2012[48] | 249 | 64 | 67.2 | Mean 8 | Female | SF-36 (PF) | ↓ |
|  |  |  |  |  | Higher age | SF-36 (PF) | ↓ |
|  |  |  |  |  | Lower SF-36 (PF) | Improvement SF-36 (PF) | ↑ |
|  |  |  |  |  | Previous hip injury | SF-36 (PF) | ↓ |
|  |  |  |  |  | Greater number of painful joint sites | SF-36 (PF) | ↓ |
|  |  |  |  |  | Worse radiological grades | Improvement SF-36 (PF) | ↑ |
| Judge, 2011[18] | 908 | 56 | 65.9 | 1 | Higher expectations | Change WOMAC | ↑ |
|  |  |  |  |  | High education | Change WOMAC | ↑ |
|  |  |  |  |  | Worse baseline pain | Change WOMAC | ↑ |
|  |  |  |  |  | Worse baseline function | Change WOMAC | ↑ |
|  |  |  |  |  | Less severe radiological change | Change WOMAC | ↑ |
| Katz, 2012[27] | 49,136 | 63 | 60% 65-75 y, 40% >75Y | 12 | Males | Revision | ↑ |
|  |  |  |  |  | Younger patients | Revision | ↑ |
| Kennedy, 2011[44] | 75 | 43 | 61 | Up to 1.3 | Higher 6MWT distance | 6MWT distance | ↑ |
| Keurentjes, 2013[20] | 445 | 63 | 66.6 | 1.5-6 | Kellgren Grade 0-2 vs 3-4 | SF-36 (PF) | ↑ |
|  |  |  |  |  | Kellgren Grade 0-2 vs 3-4 | Numeric Rating Scale Satisfaction | ↑ |
| Meding, 2000[22] | 1,015 | 55 | 67.2 | Mean 2.7 | Greater degree of cartilage space loss | Pain at 1 year | ↓ |
| Nikolajsen, 2006[30] | 1,048 | ND | ND | 1-1.5 | Females | Daily, constant pain in hip and elsewhere | ↑ |
| Nilsdotter, 2002[32] | 124 | 56 | 71 | 1 | Age >72 year | WOMAC (physical function) | ↑ |
|  |  |  |  |  | Age >72 year | SF-36 all subscales except bodily pain | ↓ |
|  |  |  |  |  | Lower WOMAC (physical function and pain) | WOMAC (physical function and pain) | ↓ |
|  |  |  |  |  | Higher SF-36 (pain) | SF-36 (pain) | ↑ |
| Nilsdotter, 2001[31] | 74 (WOMAC) | 53 | 71.2 | 1 | Higher WOMAC (physical function) | WOMAC (physical function) | ↑ |
|  |  |  |  |  | WOMAC (Pain) | WOMAC (Pain) | ↑ |
| Röder, 2007[24] | 12,925 | 50 | males 68.6 females 66,3 | Mean 4.3 | Better walking capacity | Walking capacity | ↑ |
|  |  |  |  |  | Better Flexion | Flexion | ↑ |
| Rolfson, 2009[23] | 6,158 | 57 | 69 | 1 | Charnley class C | Pain relief (VAS) | ↓ |
|  |  |  |  |  | Anxiety/ depression (EQ-5D) | Pain relief (VAS) | ↑ |
|  |  |  |  |  | Female | Satisfaction | ↓ |
|  |  |  |  |  | Anxiety/ depression (EQ-5D) | Satisfaction | ↓ |
|  |  |  |  |  | Charnley class C | Satisfaction | ↓ |
|  |  |  |  |  | Female | EQ-5D | ↓ |
|  |  |  |  |  | Anxiety/ depression (EQ5D) score 2 or 3 | EQ-5D | ↑ |
|  |  |  |  |  | Charnley class C | EQ-5D | ↓ |
| Sadr Azodi, 2008[25] | 2,106 | 0 | 30-54: 239, 55-59: 324, 60-64: 387, 65-69: 391, 70-74: 370, 75-79: 244, 80+: 151 | 3 | Obesity | Dislocation | ↑ |
| Sarasqueta, 2012[21] | 166 | 47 | 67 | 1 | Higher SF-12 (PC) | Function (WOMAC) | ↓ |
|  |  |  |  |  | Function (WOMAC) ≥76.48 | Function (WOMAC) | ↑ |
| Stickles, 2001[26] | 592 | 56 | 68.9 | 1 | Higher BMI | Ascending and descending stairs | ↓ |
| Street, 2005[33] | 236 | ND | 67.1 | 1 and 2 | Knee pain (vs hip and thigh pain) | HHS | ↓ |
|  |  |  |  |  | Knee pain (vs hip pain) | WOMAC | ↑ |
|  |  |  |  |  | Knee pain (vs hip and thigh pain) | SF-36 (physical function, vitality, social function and mental health at 1 and 2 year f-up and role physical at 2 year f-up | ↓ |
| Tanaka, 2010[45] | 43 | 100 | 59.7 | 1 | Severe stage hip OA (radiological) | Gait improvement | ↓ |

§ Follow-up moments ≥1 year, when analyses were performed

† Based on both THA and TKA population

ND Not described

*Direction according to scale of the instrument (e.g. VAS pain, VAS Satisfaction, WOMAC: lower scores indicate better outcomes, SF-36, HOOS, HHS etc.: higher scores indicate better outcomes).

*BIPQ: The Brief Illness Perception Questionnaire; HHS: Harris Hip Score; SF-12: 12-item Short Form Health Survey; OHS: Oxford Hip Score; DEPCAT: Deprivation Categories; SF-36: 36-item Short Form Health Survey; PF: Physical Functioning; RP: Physical Role; BP: Bodily Pain; GH; General Health; VT: Vitality; SF: Social Functioning; RE: Role-Emotional; MH: Mental Health; MCS: Mental Component Summary score; PCS: Physical Component Summary score; BMI: Body Mass Index; HOOS: Hip disability and Osteoarthritis Outcome Score; ADL: Activities of Daily Living; QOL: Quality Of Life; EQ-5D: EuroQol 5 Dimensions WOMAC: Western Ontario & McMaster Universities Osteoarthritis Index; VAS: Visual Analogue Scale; 6MWT: 6 Minute Walk Test; ROM: Range Of Motion; ESSI: The ENRICHD Social Support Instrument; HADS: Hospital Anxiety and Depression Scale; OA: OsteoArthritis*
